# Activation of kappa opioid receptor suppresses post-traumatic osteoarthritis via sequestering STAT3 on the plasma membrane

**DOI:** 10.1186/s12964-024-01709-4

**Published:** 2024-06-18

**Authors:** Haixia Liu, Renhuan Huang, Ziang Zhuo, Xinru Zhang, Ling Wu, Zhen Guo, Fuping Wen, Liwei An, Hang Yuan, Yiming Zhang, Yuanzhi Xu

**Affiliations:** 1 Department of Stomatology, Shanghai Tenth People’s Hospital, School of Medicine, Tongji University, Shanghai, China; 2 iView Therapeutics, Inc., Cranbury, NJ USA

**Keywords:** Osteoarthritis, Kappa opioid receptor, STAT3, TNF-α

## Abstract

**Objective:**

Kappa opioid receptor (KOR) signaling is involved in joint development and inflammation in Osteoarthritis (OA), while the biochemical mechanism remains unclarified. This study aims to investigate downstream molecular events of KOR activation, to provide novel perspectives in OA pathology.

**Methods:**

U50,488H, a selective KOR agonist, was intra-articularly injected in mice upon destabilization of the medial meniscus (DMM) as OA models, with PBS injection as control. The behavioral and histological evaluation was assessed by hot plate test and red solid green staining, respectively. Alterations in mRNA and protein expression were assessed by RNA-seq, RT-qPCR, immunohistochemistry and western blotting (WB) in chondrocytes treated with TNF-α or TNF-α + U50,488H. Proteins interacted with KOR were explored using proximity labeling followed by mass spectrometry and then testified by co-immunoprecipitation (Co-IP) assay and immunofluorescence (IF).

**Results:**

OA-induced pain was reduced and cartilage degeneration was alleviated upon KOR activation in DMM mice. In chondrocytes, activation of KOR reversed the upregulation of MMPs, IL-6, IL-1β and phosphorylated(p-) STAT3, stimulated by TNF-α, while the expression of NF-κB, MAPKs and AKT signaling weren't reversed. RNA-seq and IF results presented that KOR activation evidently reduced STAT3 nuclear translocation in chondrocytes upon TNF-α stimuli. The reduction may be resulted from the binding of KOR and STAT3 in the plasma membrane, revealed by proximity labeling and Co-IP results.

**Conclusions:**

KOR activation protects cartilage from OA, and this protective effect is mainly exerted via sequestering STAT3 on the plasma membrane, resulting in inactivation of STAT3-dependent immune responses which otherwise contributes to OA.

**Supplementary Information:**

The online version contains supplementary material available at 10.1186/s12964-024-01709-4.

## Introduction

Osteoarthritis (OA) is a prevalent degenerative disease, globally affecting 500 million people, especially the elderly [1]. Patients with OA mainly suffer from chronic pain and restricted joint function, which leads to poor life quality over the course of disease [2]. The pathology of OA is typically characterized by inflammatory deterioration of articular cartilage, synovial tissue, and subchondral bone [2]. Articular cartilage usually gets severely degraded as the disease progresses [2]. Current therapeutic approaches are limited to physical therapies, nonsteroidal anti-inflammatory drugs (NSAIDs) and glucosamine in the early stage and surgical treatment for severe cases [3]. The development of a more effective therapy is urgently needed which should relieve symptoms meanwhile reversing the disease. Therefore, a comprehensive understanding of the molecular mechanism underlying OA pathogenesis is of significance.

Kappa opioid receptor (KOR) is one of the classic opioid receptor family(mu opioid receptors, delta opioid receptors, kappa opioid receptors and opioid receptor–like 1 receptors), which belongs to the G protein-coupled receptor (GPCR) superfamily [4]. KOR is widely expressed in the membrane of multiple types of cells including the central/peripheral nervous system, gastrointestinal tract, immune system, joint-forming cells, etc. [5]. In recent decades, KOR agonists are promising targets in developing non-addictive and anti-nociceptive analgesics, with less side-effects such as respiratory depression, compared with other opioids [6]. Besides pain-relieving effects, previous researches evidence that activation of KOR receptors is effective in the treatment of inflammatory bowel disease, subarachnoid hemorrhage, pruritus, multiple sclerosis, Alzheimer’s disease, and joint inflammation [7–11]. In addition, KOR has also been reported to play an essential role in OA [12], but underlying mechanisms have not been fully described. Specifically, our previous results showed that KOR knockout mice presented accelerated cartilage degeneration compared with wild type (WT) mice after injury [13]. Weber et al. further found that KOR agonist JT09 decreased cartilage degeneration in osteoarthritis by inhibiting Hedgehog signaling in both human chondrocytes and rat model [14]. In a recent study targeting the macrophages in OA, the in vivo and in vitro results revealed that activation of KOR by U50,488H, suppressed macrophage M1 polarization through inhibiting the translocation of NF-κB into the nucleus, thus the expression of inflammatory cytokines TNF-α and IL-6 secreted by macrophages was reduced [15]. Differently, in another study of rheumatoid arthritis model treated with naltrexone (NTX), an antagonist of KOR and DOR receptors, decreased erosion of joint cartilage and bone was reported, through inhibition of TLR4/NF-κB pathway, and NTX reduced the expression of the pro-inflammatory cytokines (TNF-α, IL-6, IL-12, IL-17) while increased the expression of the anti-inflammatory cytokine (IL-10) [16]. Thus, KOR and its downstream signaling may exert varied anti-inflammatory effects, depending on disease conditions and molecular characteristics of the ligands, which leads to further exploration.

This study aims to further investigate the down-stream reactions of KOR activation in OA mouse models, in order to provide a novel insight on the treatment of OA and the clinical potential of KOR-targeted medication.

## Materials and methods

### DMM mouse model of OA

DMM model was established in 10-week-old C57BL/6 mice as previously described [17] to induce post-traumatic OA. Sham surgery was performed by incision of the cutaneous and muscular planes at baseline (Control group, *n* = 9). Mice were randomly assigned to receive intra-articular injection of U50,488H (TOCRIS, USA) (2.5 mg/kg once a week) (DMM + U50 group, *n* = 12) or PBS as a control (DMM group, *n* = 12) for 8 weeks from the day after the operation. Eight weeks after surgery, the knees were harvested and further analyzed. All animal experiments in this study were accomplished in accordance with the National Institutes of Health Guidelines for the Care and Use of Laboratory animals and approved by the ethical guidelines of Animal Ethics Committee of Tenth People’s Hospital, Shanghai, China (approval no. SHDSYY-2016-X0032).

### Hot plate test

Mice were transferred to the test room at least 30 min before experiment, and then placed on the hotplate at 55℃ one at a time (DLAB, China). The latency period for hind limb response (e.g., shaking, jumping, or licking) was recorded as response time. Each trial lasted maximumly for 45 s. The mouse was removed from the hotplate immediately after a response was recorded [18].

### Cell culture

Primary chondrocytes were isolated from 4-week mice. Briefly, the cartilage tissue from costa was minced and digested with 1 mg/ml collagenase II (Worthington, USA) in Dulbecco’s modified Eagle’s medium (DMEM, Gibco Life Technologies, USA) containing 10% fetal bovine serum (FBS, Gibco Life Technologies, USA) and 2% penicillin/streptomycin (Gibco Life Technologies, USA) at 37 °C for more than 16 h. Pellets were resuspended in DMEM and cultured in DMEM containing 10% FBS, and 1% penicillin/streptomycin until confluence. The chondrogenic cell line ATDC5 and 293 T cells were cultured in DMEM containing 10% FBS, and 1% penicillin/streptomycin.

Confluent monolayers of chondrocytes were stimulated with or without tumor necrosis factor α (TNF-α, R&D Systems, USA), U50,488H and Stattic (MedChemExpress, USA), a nonpeptidic small molecule that selectively inhibits STAT3 [19] and norbinaltorphimine (nor-BNI, aladdin, China), the kappa opioid receptor antagonist [20]. At the end of culture, cells were collected for detection. All experiments were performed in duplicate.

### Western blot

Cell protein (20–50 μg) from cells was separated by sodium dodecyl sulfate polyacrylamide gel electrophoresis (SDS-PAGE). Gels were transferred onto polyvinylidene fluoride (PVDF) membranes (Amersham Biosciences, USA) and then the PVDF membranes were blocked 1 h in 5% non-fat milk (Beyotime Biotechnology, China) diluted in PBS (Beyotime Biotechnology, China) containing 0.1% Tween 20 (Beyotime Biotechnology, China), with gentle agitation at room temperature (RT). Membranes were incubated with antibodies P65, p-P65, p-RelB, RelB, p-IκBα, IκBα, p-P105, P105, STAT3, p-STAT3, p-P38, P38, p-JNK, JNK, p-ERK, ERK (1:1000, Cell Signalling Technology, USA), KOR, MMP3, MMP13 (1:1000, Abcam, USA), TurboID (1:2000, Agrisera, Sweden), Flag (1:5000, sigma, Germany) diluted in 5% non-fat milk at 4 °C overnight with gentle agitation. GAPDH (1:5000, ProteinTech, USA) was used as a loading control. Following three times washes, membranes were incubated with appropriate horseradish peroxidase conjugated secondary antibodies (1:1000, ProteinTech, USA) for 1 h at RT. The signals were detected using Enhanced ChemiLuminescence (Beyotime Biotechnology, China), and density of the bands was analyzed using Image J.

### RT-qPCR

Total RNA was isolated using Trizol reagent (Invitrogen, USA) following the manufacturer’s protocol. Prime Script RT reagent Kit with gDNA Eraser (Takara Bio, Japan) was used to synthesize cDNA from 1 μg RNA. The real-time RCR reaction was amplified using SYBR Premix Ex Taq II (Takara Bio, Japan) and an ABI 7500 Real-Time PCR System (Applied Biosystems, USA). The specific primers to the following mRNA were used: *Gapdh, Mmp3, Mmp13, Adamts4, Adamts5, Il-6 and Il-1β*. The primer sequences were listed below:

### Gapdh

Forward: 5′-GGGAAGCCCATCACCATCTT-3′

Reverse: 5′-GCCTCACCCCATTTGATGTT-3′,

### Mmp3

Forward: 5′-GTGGATCCTGGCAACCACA-3′

Reverse: 5′-CAGCACTCGCAGTCTGAGTT-3′,

### Mmp13

Forward: 5′-CACACCTCACCATCAATGCTGC-3′

Reverse: 5′-GAAGGGTTGGACACCTGAATGC-3′,

### Adamts4

Forward: 5′-AGCCACAGCAGCCTCAGAGAC-3′

Reverse: 5′-AGCCACAGCAGCCTCAGAGAC-3′,

### Adamts5

Forward: 5′-TCAGACTTGGTGGAGGCGTAGG-3′

Reverse: 5′-AGGCGGATGTGGTTCTCAATGC-3′,

### Il-6

Forward: 5′-CACTCCCACCCTGAGATTTGT-3′

Reverse: 5′-CATCGTCTGCACGGTTCTG-3′,

### Il-1β

Forward: 5′-AAGTCTGTCCTTCCGCAGTC-3′

Reverse: 5′-TGAAGAAAGTTATCTGGGTAGCTCA-3′.

Relative gene expression was calculated using the comparative 2-ΔΔCt method. Each measurement was assessed in triplicate. The gene expression ratio is shown as the mean  ±  SD from three independent experiments.

### Red solid green staining

Mouse tissues were fixed in 4% paraformaldehyde for 48 h, decalcification with 10% EDTA solution, dehydrated with ethanol, and embedded in paraffin using routine procedures. A microtome (Leica, Germany) was used to cut 5 μm sections. Slides were then deparaffinized and stained for sulfated GAG with Alcian blue and safranin O. The staining was performed to assess morphology. The degradation of GAGs in cartilage matrix was assessed after safranin O staining of sections following OARSI recommendations [21]. Three sections from every mouse specimen were scored by 3 blinded researchers. The average numbers from 3 researchers were used to calculate the final score.

### Immunohistochemistry and immunofluorescence

For immunohistochemistry (IHC), paraffin sections were processed using routine procedures. Sections were incubated with antibodies ACAN (Aggrecan, 1:100, Abcam, USA), COMP (Cartilage oligomeric matrix protein, 1:500, Abcam, USA), COL2 (1:500, Abcam, USA), MMP3 (1:50, Abcam, USA), MMP13 (1:100, Abcam, USA) diluted in 5% non-fat milk at 4 °C overnight. The antibodies against specific antigens were used, followed by incubation of HRP-conjugated secondary antibodies and then visualized by a DAB Peroxidase Substrate Kit (Bioworld, USA).

### RNA-sequencing (RNA-Seq)

Primary chondrocytes were treated with TNF-α or TNF-α + U50,488H for 24 h. RNA was extracted from three biological replicates. RNA was extracted from the rat chondrocytes and cartilage tissue using Trizol reagent. cDNA library preparation and sequencing were on the Illumina Hiseq2000 platform by Shanghai Applied Protein Technology (China). The FPKM method was used to determine gene expression. We applied the DESeq2 algorithm to identify the differentially expressed genes. Significant analysis was performed using the P-value. Differentially expressed genes were considered as those with a fold change > 2 or fold change < 0.5, P-value < 0.05. Pathway analysis was used to identify the significantly influenced pathways, in which the differentially expressed genes were selected according to the Kyoto Encyclopedia of Genes and Genomes (KEGG, http://www.kegg.jp/) database [22]. Enrichment analysis was performed on STAT3 pathway by Gene Set Enrichment Analysis (GSEA) [23].

### Co-immunoprecipitation (Co-IP) assay

The experiment was operated referring to protocol [24]. ATDC5 cells were treated with TNF-α or TNF-α + U50,488H for 24 h, and were lysed with lysis buffer with 1 μM PMSF (Beyotime Biotechnology, China). And then cells were incubated with protein A/G PLUS-Agarose (Santa Cruz, USA) and 5 μg antibody KOR (Abcam, USA) overnight at 4℃. Washed the beads with PBS 5 times and samples were used for WB.

### Plasmid constructions and cell transfection

The full sequence of NotI-KOR-MluI was synthesized by Beijing Genomics institution (China). The product was cleaved with restriction enzyme NotI (Biolabs, USA) and MluI (Biolabs, USA), and polymerized with the cleaved vector of C-terminally tagged pLV-TurboID by T4 ligase (Thermo Fisher, USA). PEI (Yeason, China) was used for cell transfection followed by the manual.

### Mass spectrometry and protein identification

The pLV-KOR-TurboID plasmid was transfected into 293 T cells for 24 h. Then cells were stimulated with biotin (Sigma-Aldrich, USA) and TNF-α and U50,488H for 12 h. After that 293 T cells were collected using a scraper and lysed in lysis buffer. The cell lysate was incubated with streptavidin beads (Smart-Lifesciences, China) overnight. Washed the beads with PBS 5 times and samples were for mass spectrometry and protein identification. SDS-PAGE was performed and protein bands were excised from the gel and then analyzed on LCMSMS (nanoLC-QE) by Shanghai Applied Protein Technology (China).

### Image acquisition and quantification

All images including bright field and fluorescence were made with microscope (Leica, Germany). Quantification of positively stained cells by immunohistochemistry was performed with ImageJ.

### Statistical analysis

Statistical analysis was performed using one -way ANOVA followed by Tukey honest significant difference post-hoc test (GraphPad Prism software) to compare more than 2 groups, or 2-tailed Student’s *t* test to compare 2 groups. *P* values less than 0.05 is considered to be significant.

## Results

### KOR activation decelerates the progression of osteoarthritis

**Fig. 1:**
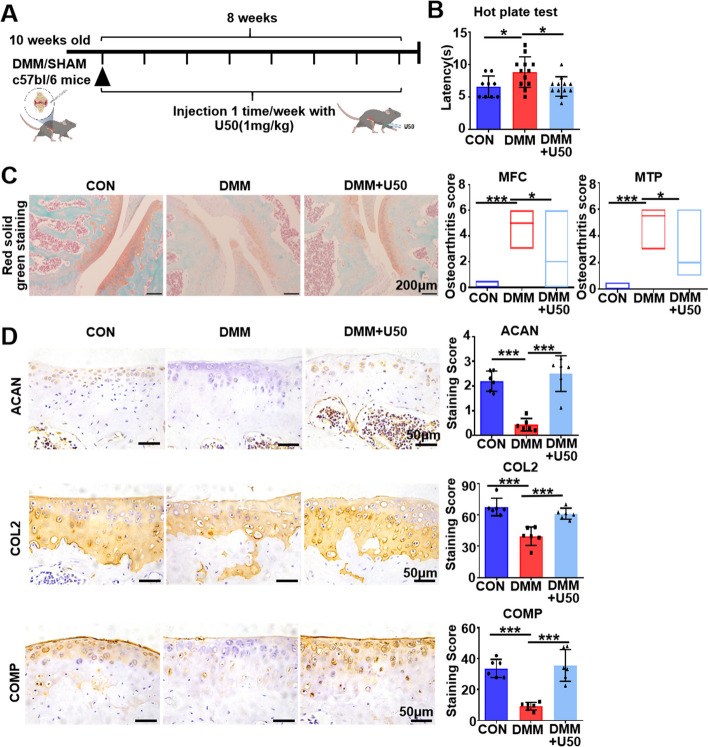
KOR activation decelerates the progression of osteoarthritis. **A** The schematic diagram of the in vivo experiment. **B** Results of the hot plate assessment (*n* = 9–12 mice each group). **C** Results of the Red Solid green dyeing and quantification in items of medial femoral condyle (MFC) and medial tibial plateau (MTP) according to OARSI (*n* = 9–12 mice each group). Scale bar: 200 μm. **D** Immunochemical staining and quantification results of ACAN, COL2 and COMP, respectively (*n* = 9–12 mice each group). Scale bar: 50 μm. One-way ANOVA, *P < 0.05, **P < 0.01, ***P < 0.001. U50 is short for U50,488H. Data are mean ± SD or median and defined ranges

To investigated the effects of KOR activation on OA, we first compared the OA progression status in mice [13, 25] co-treated with or without U50,488H after DMM operation (Fig. 1A). Compared to the sham group, the hotplate nociception analysis revealed a significantly delayed response to pain stimulus in the DMM group (*P* = 0.0287) (Fig. 1B). Interestingly, treatment with U50,488H efficiently rescued such delayed response (*P* = 0.0187) (Fig. 1B). Eight weeks after surgery, the knees were harvested and further analyzed. Knee joints were collected at 8 weeks after surgery for histological analysis (Fig. 1C). To assess the severity of cartilage damage in OA, Osteoarthritis Research Society International (OARSI) score was applied to analyze red solid green staining results [21]. Evident articular cartilage damage was observed in the DMM-operated mice (MFC: *P* < 0.001; MTP: *P* < 0.001) and OA lesions were significantly less severe in the DMM + U50,488H group than that in the DMM group (MFC: *P* = 0.0383; MTP: *P* = 0.0313) (Fig. 1C). Expression levels of cartilage-specific protein aggrecan (ACAN), type 2 collage (COL2) and cartilage oligomeric matrix protein (COMP) were investigated in mouse cartilage tissue. The results of IHC indicated that the expression of ACAN, COL2 and COMP were restrained in the DMM group (*P* < 0.001; *P* < 0.001; *P* < 0.001, respectively), but recovered in the DMM + U50,488H group (*P* < 0.001; *P* < 0.001; *P* < 0.001, respectively) (Fig. 1D). All these in vivo data indicated that the local activation of KOR by U50,488H attenuated post-traumatic osteoarthritis in mice.

### KOR activation reduces the expression of cartilage-catabolic markers

**Fig. 2:**
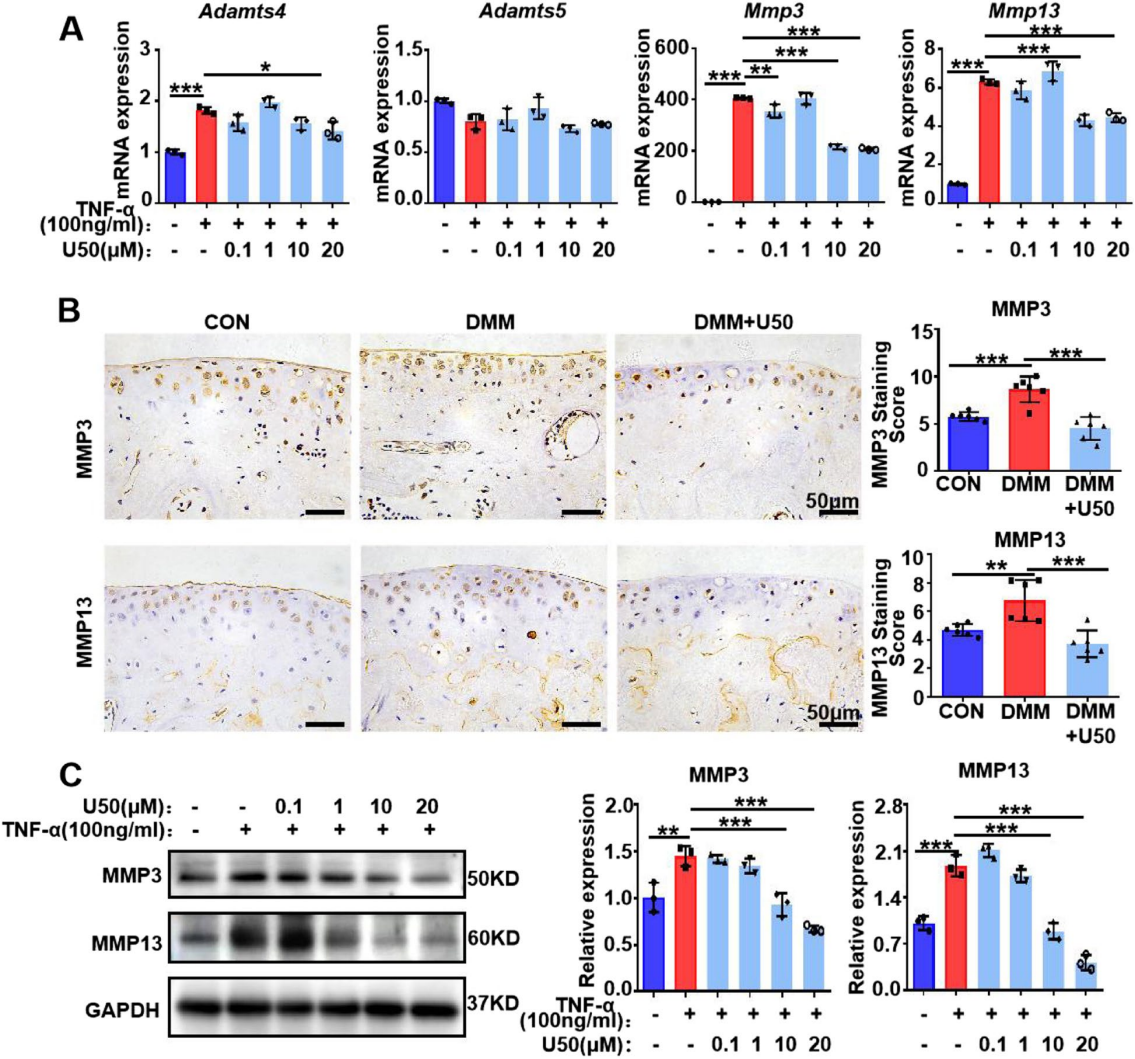
KOR activation reduces the expression of cartilage-catabolic markers. **A** Genes expression of cartilage-degrading enzymes in the RT-qPCR results. **B** Immunohistochemical results and quantification of MMP3 and MMP13 in mice (*n* = 6 mice each group). Scale bar: 50 μm. **C** Expression and quantification of MMP3 and MMP13 in the WB results. One-way ANOVA. *P < 0.05, **P < 0.01, ***P < 0.001. U50 is short for U50,488H. Error bars indicate ± SD

Then, we evaluated the changes in the expression level of disintegrin and metalloproteinase with thrombospondin motifs (Adamts) as well as matrix metalloproteinase (MMPs) and major anabolic and phenotypic genes in primary mouse chondrocytes. To this end, cells were first cultured in 100 ng/ml TNF-α, to mimic cartilage degradation in vitro, together with increased dose of U50,488H. After 24 h, the U50,488H (10 μM and 20 μM), as expected, significantly reduced the mRNA levels of catabolic markers, such as *Mmp3*, *Mmp13* and *Adamts 4* in TNF-α-treated chondrocytes, especially *Mmp3* and *Mmp13* (*P* < 0.001) (Fig. 2A).

Next, IHC and WB were performed to detect the protein level of MMP3 and MMP13 in primary mouse cartilage tissue and chondrocytes. The results showed that U50,488H dose-dependently reduced MMP3 and MMP13 upon TNF-α stimulation, and it also down-regulated expression of MMP3 and MMP13 comparing with the DMM group (Fig. 2B-C). Therefore, activation of KOR may exert protective effects on chondrocytes through reducing the expression of MMPs while it had minimal influence on anabolic genes.

### KOR activation attenuates the inflammatory responses in chondrocytes

**Fig. 3:**
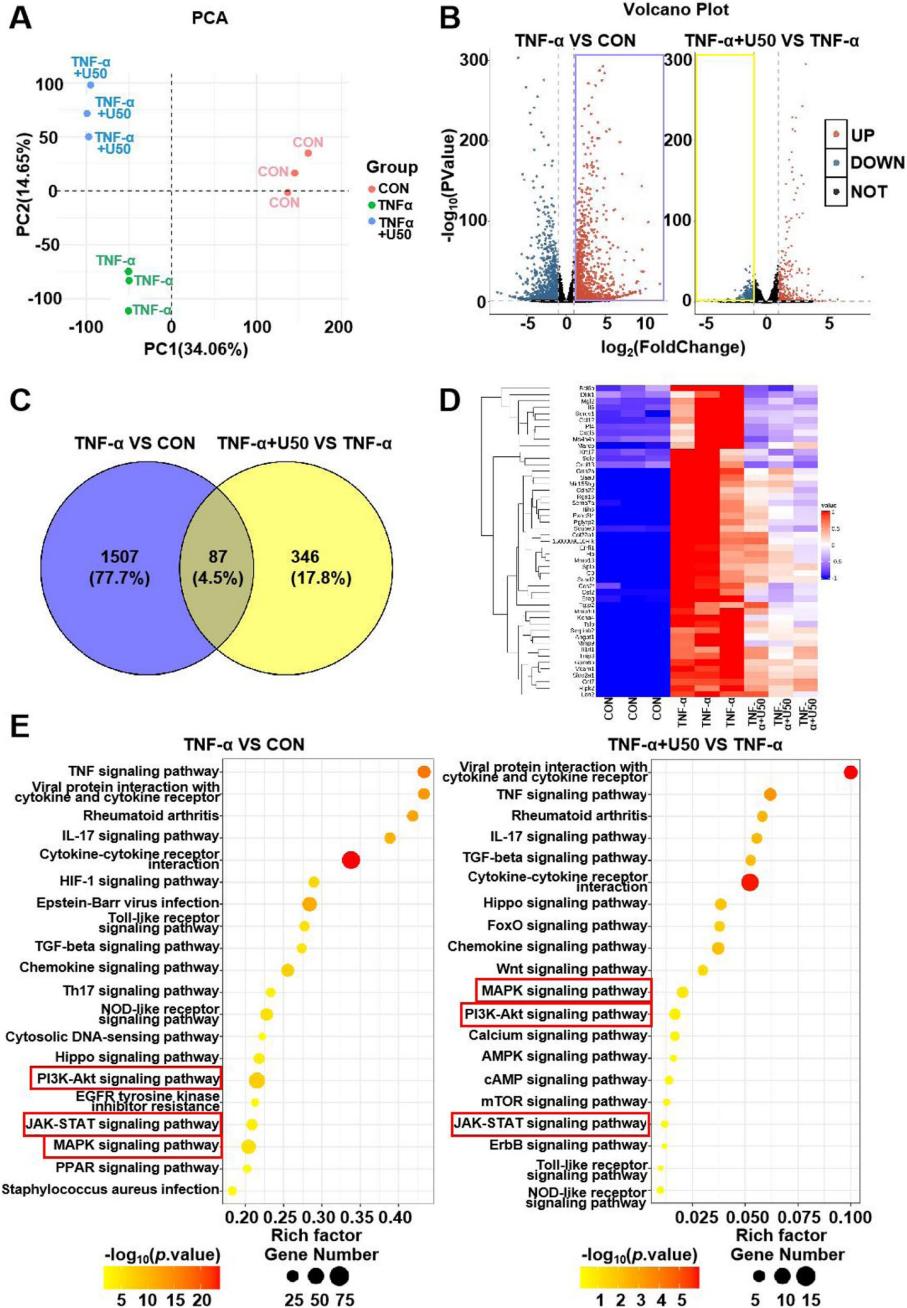
Activation of KOR down-regulated the inflammatory reactions in chondrocytes upon TNF-α stimuli. **A** PCA analysis result of three groups. **B** Differentially expressed genes in the three groups presented in the volcano plot. We mainly focused on the proteins up-regulated on TNF-α stimuli and reversed by KOR activation as shown in the purple and yellow box, respectively. **C** Venn diagram showed the proteins up-regulated on TNF-α stimuli and reversed by KOR activation through paired comparison. **D** Heatmap shows the quantification of differentially expressed proteins. Results from three biological replicates. (E) KEGG analysis results showed the pathway enrichment of differentially expressed proteins in the three groups. U50 is short for U50,488H. TNF-α VS CON represented TNF-α group compared to the control group and TNF-α + U50 VS TNF-α represented TNF-α + U50,488H group compared to the TNF-α group

To further clarify the molecular mechanisms of KOR activation-mediated OA alleviation, we treated primary chondrocytes with TNF-α (100 ng/ml) together with or without 20 μM U50,488H for 24 h before harvesting for RNA-sequencing. The principal component analysis (PCA) showed a high degree of similarity in the samples from the biological replicates (Fig. 3A). Among the differentially expressed genes (fold change > 2 and *P* < 0.05), 1594 genes were found to be up-regulated in TNF-α group compared to the control group, and 433 genes were down-regulated in TNF-α + U50,488H group than TNF-α group, as presented in the volcano plot (Fig. 3B). Based on variation fold, we screened 49 (fold change > 3) out of 1594 genes that were up-regulated in TNF-α group meanwhile down-regulated in TNF-α + U50,488H group, as shown in the Venn diagram in Fig. 3C and the heatmap in Fig. 3D. Heatmap results showed the top 49 genes screened from 26,025 varied genes including multiple inflammation-related genes and MMPs (Fig. 3D). Results of KEGG pathway analysis of these up-regulated genes by TNF-α and down-regulated genes by U50,488H displayed respectively that activation of KOR down-regulated the inflammation-related pathways in chondrocytes stimulated by TNF-α (Fig. 3E). Among the pathways and genes of interest, we performed validation tests on the expression of inflammatory cytokines and NF-κB, AKT, MAPK and STAT3 pathways that may respond to KOR activation.

According to previous literature, STAT3 and NF-κB transcription factor, as well as MAPK and AKT were abnormally activated in OA and participate in many OA-associated events [19, 26–28].

### KOR activation limits STAT3-mediated immune responses in chondrocytes

**Fig. 4:**
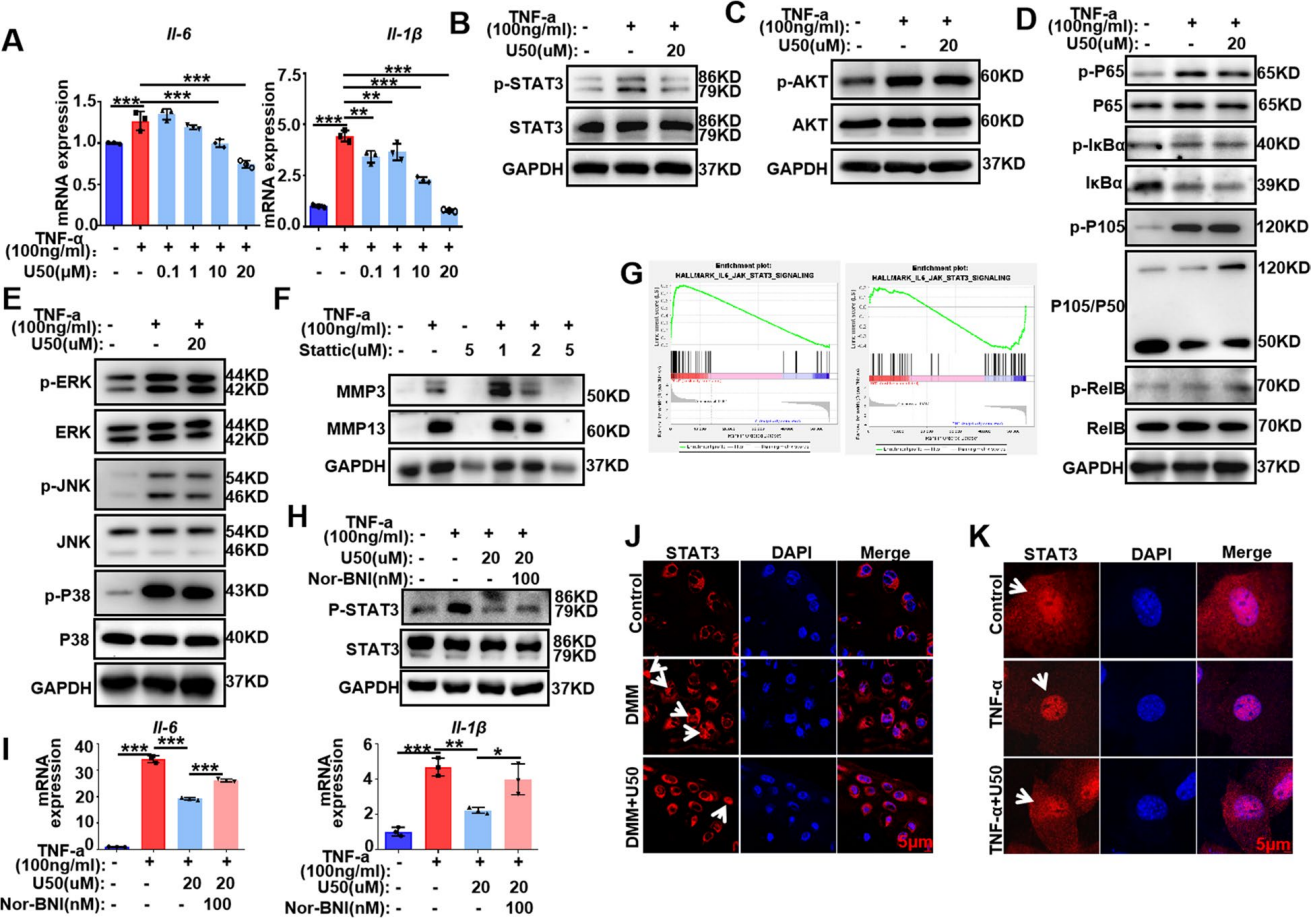
KOR activation limits STAT3-mediated immune responses in chondrocytes. **A** RT-qPCR results showed the expression of *IL-6* and *IL-1β*. One-way ANOVA analysis, *P < 0.05, **P < 0.01, ***P < 0.001. Error bars indicate ± SD. **B** WB results of STAT3 signaling pathway. **C** WB results of AKT signaling pathway. **D** WB results of NF-κB signaling pathway. **E** WB results of MAPK signaling pathway. **F** WB tested the impacts of stattic, a selective STAT3 inhibitor, on expression of MMP3 and MMP13. **G** Enrichment of STAT3 pathway by GSEA. **H** WB results of STAT3 signaling pathway. **I** RT-qPCR results showed the expression of *IL-6* and *IL-1β*. One-way ANOVA analysis, *P < 0.05, **P < 0.01, ***P < 0.001. Error bars indicate ± SD. **J** IF tested the location of STAT3 in mice. **K** IF tested the location of STAT3 in primary chondrocytes. Scale bar: 5 μm. STAT3 (red), DAPI (blue), U50 is short for U50,488H

To verify the results of RNA-seq, the expression level of pro-inflammatory cytokines was investigated in chondrocytes treated with TNF-α or TNF-α + U50,488H for 24 h. RT-qPCR results demonstrated that U50,488H could significantly suppress, via a dose-dependent manner, the expression of pro-inflammatory cytokines *Il-6* and* Il-1β* (Fig. 4A).

According to the results of KEGG analysis (Fig. 3E), we next applied western blotting analysis to investigate the inflammation-related pathways in chondrocytes treated with TNF-α (100 ng/ml) or TNF-α + U50,488H (20 μM), or PBS as control for 30 min. Consistent with previous reports, TNF-α treatment markedly enhanced the phosphorylation of STAT3 (STAT3 signaling), AKT (AKT signaling), P65, P105 and (NF-κB pathway), as well as ERK, JNK, P38 in MAPK pathway. Nevertheless, co-treatment with U50,488H specifically rescued the phosphorylation level of STAT3 on TNF-α stimulation but not NF-κB, MAPK or AKT signaling (Figs. 4B-E). Thus, KOR activation may play a role in relation with STAT3 pathway, a typical signal pathway involved in OA progression [19]. In line with this, we next treated the cells with stattic, a selective inhibitor of STAT3 [29], and found that it could dose-dependently reduce the expression of Mmp3 and Mmp13 (Fig. 4F) in TNF-α treated chondrocytes.

Gene Set Enrichment Analysis (GSEA) was used to analyze the data of RNA-sequencing, and the results showed that TNF-α stimulation up-regulated the STAT3 pathway which was reversed by administration of U50,488H in chondrocytes (Fig. 4G). To further prove that U50,488H inhibits inflammation by activating KOR and inhibiting STAT3 signaling as well, nor-BNI (100 nM), an antagonist of KOR, was used to verify whether it could block the effect of U50,488H on chondrocytes or not. The results showed that the nor-BNI could reverse the phosphorylation of STAT3 signaling inhibited by U50,488H in ATDC cells treated for 30 min, and could prevent the inhibitory effect of U50,488H on inflammation in ATDC cells treated for 24 h. (Fig. 4H-I).

We also investigated the location of the STAT3. Immunofluorescence for STAT3 was performed in chondrocytes from mouse DMM model and primary chondrocytes. Results presented that the expression of STAT3 in nucleus was higher in the DMM group than that in the DMM + U50,488H group and the sham control group (Fig. 4J). Similarly, increased expression of STAT3 was also observed in the nucleus of chondrocytes in TNF-α-treated group compared to the control. More STAT3 in the cytoplasm was presented in the TNF-α + U50,488H group than the TNF-α group (Fig. 4K). It’s indicated that KOR activation may inhibit the nucleus translocation of STAT3, thereby decreasing the expression of inflammatory cytokines and alleviating inflammation reactions in chondrocytes.

Thus, KOR activation most likely exerted the protective effect on cartilage from OA through restraining STAT3 signaling pathway.

### KOR interacts with and sequesters STAT3 on the plasma membrane

**Fig. 5:**
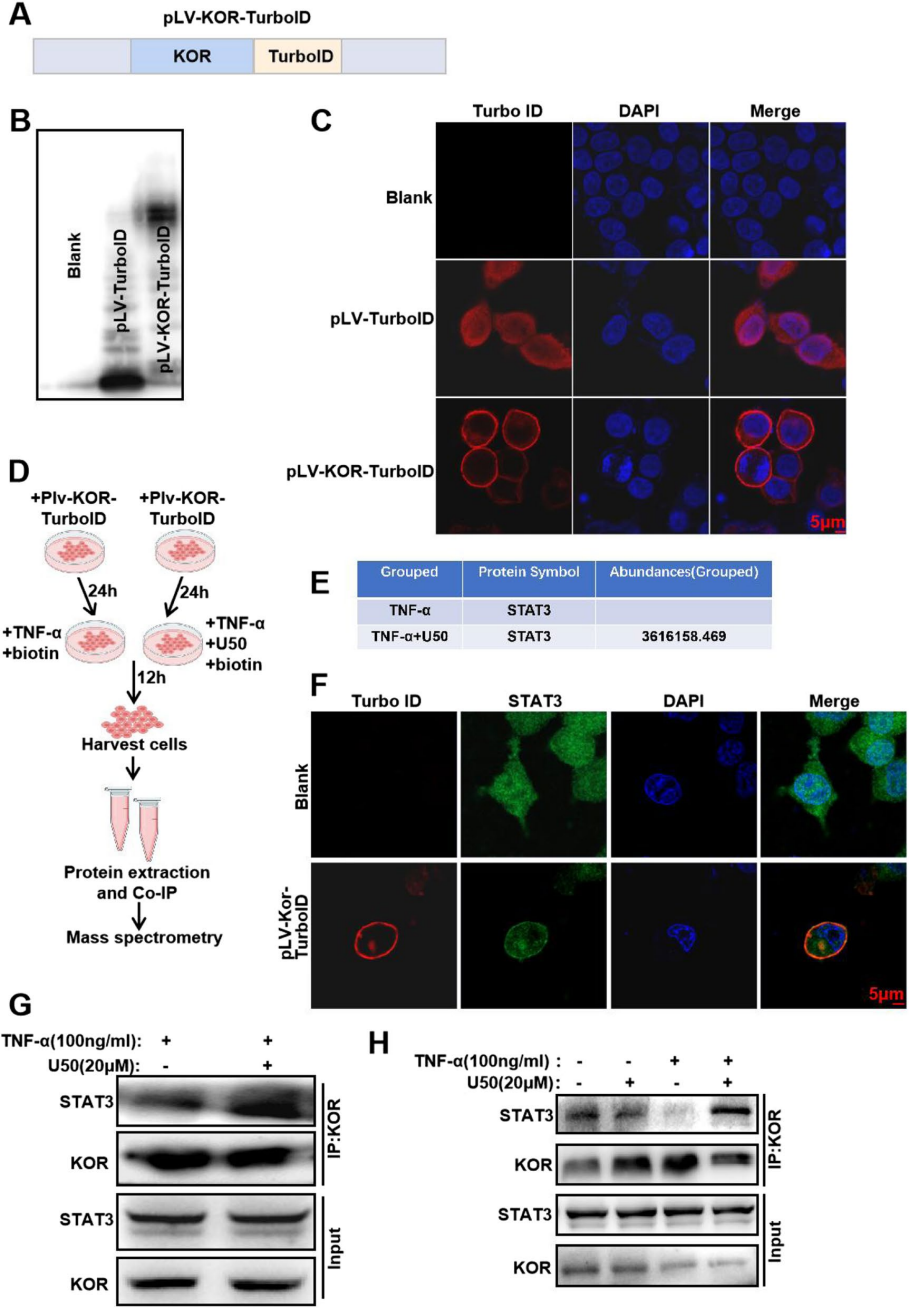
KOR interacts with and sequesters STAT3 on the cell plasma. **A**. The sketch map of plasmid construction. Chondrocytes 293 T cells and ATDC5 cells were transfected with pLV-KOR-TurboID-expression plasmid for 48 h; then expression of TurboID protein were detected using (**B**) western blotting and (**C**) immuno-fluorescence assay, respectively. Scale bar: 5 μm. **D** Workflow for sample preparation and mass spectrometry analysis. **E** The results of mass spectrometry detection of STAT3 expression. Scale bar: 5 μm. **F** After plasmid transfection in 293 T cells for 48 h, immunofluorescence was used to detect changes in the of STAT3 localization. ATDC5 cells (**H**) and transfected 293 T cells (**G**) and were cultured with TNF-α or TNF-α + U50,488H for 24 h and Co-IP was used to confirm the binding of KOR and STAT3. U50 is short for U50,488H

**Fig. 6:**
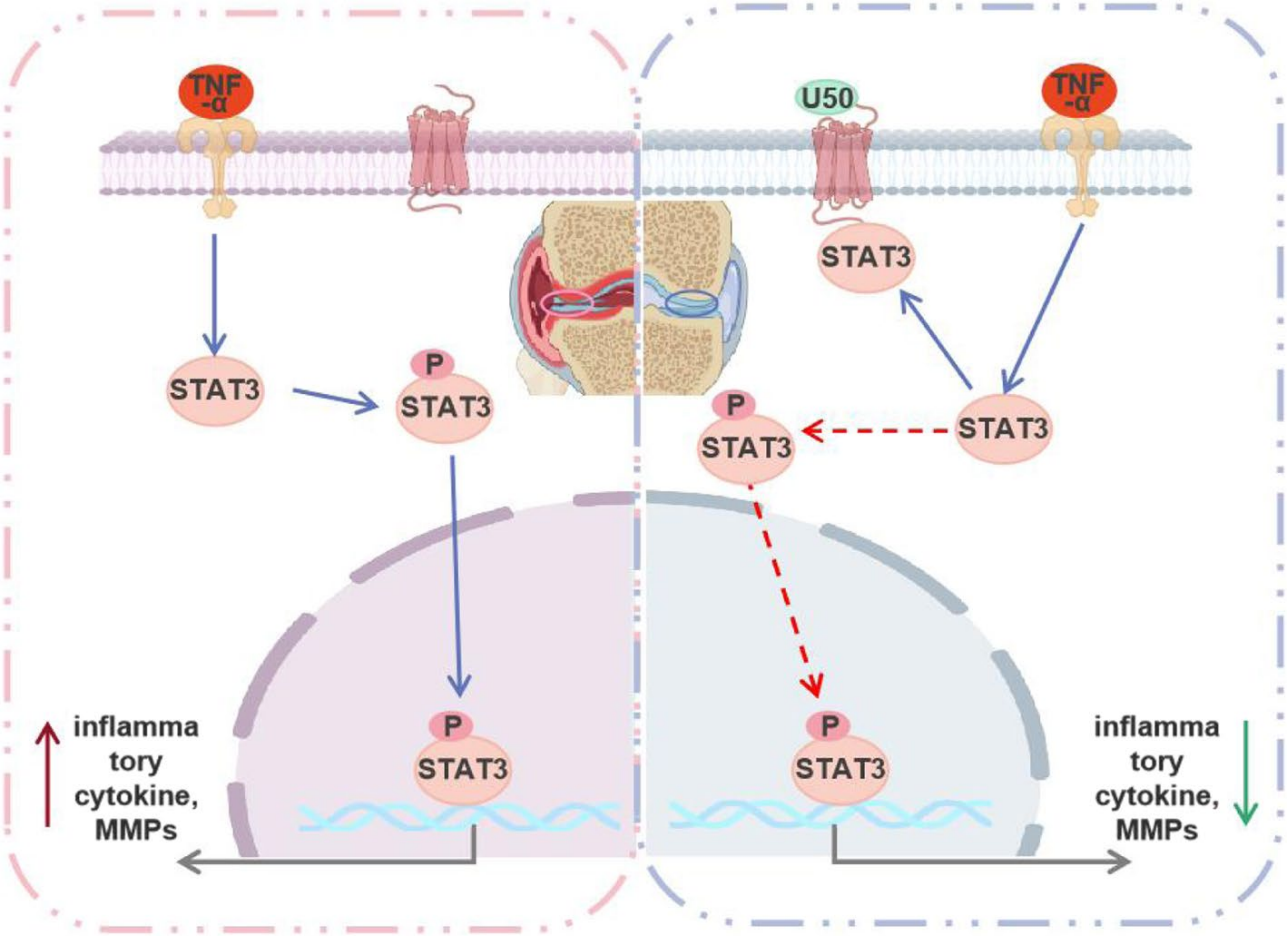
Schematic diagram demonstrating that activation of KOR restrains the STAT3 pathway via binding to STAT3 and thereby protects chondrocytes against OA. ↑: up-regulation and ↓: down-regulation (Created with BioRender. com.). U50 is short for U50,488H

To find out the relation between KOR activation and the STAT3 pathway inactivation, we next applied the recently developed proximal labeling strategy [30] to explore possible protein interactions of KOR. To this end, we constructed the lentiviral based expression plasmid of pLV-KOR-TurboID to find KOR-related interactors of proteins of interest (Fig. 5A). First, the molecular weight and location of KOR-TurboID showed that the plasmid was correctly expressed in 293 T cells, as verified by WB and IF, respectively (Fig. 5B-C). Next, we transfected 293 T cells the plasmid of KOR-TurboID transferring for 24 h before addition of biotin and TNF-α with U50,488H or not. Proteins biotinylated by TurboID were then enriched with streptavidin beads and identified by mass spectrometry (Fig. 5D). Among candidate proteins that may interact with KOR, we confirmed that STAT3 could bind with KOR (Fig. 5E). The abundance of STAT3 in TNF-α + U50,488H group was higher than TNF-α group (Fig. 5E). We verified this result with IF (Fig. 5F) and WB (Fig. 5G), which revealed that activation of KOR by U50,488H promoted the binding of STAT3 and U50,488H. In the end, we verified the binding of STAT3 and U50,488H in chondrocytes (ATDC5 cells). The result showed that STAT3 could bind with KOR and activation of KOR by U50,488H could raise the number of STAT3 binding with KOR in chondrocytes upon TNF-α stimulation (Fig. 5H). Thus, activation of KOR mediates STAT3 pathway to protect chondrocytes in OA. Overall, our data demonstrated that activation of KOR promoted binding with STAT3 which decreased the phosphorylation of STAT3 and helped to protect chondrocytes against inflammation of OA (Fig. 6).

## Discussion

Pain management is one of the major therapeutic approaches for OA [31]. KOR is one of the classic opioid receptors in pain modulation, as a promising target for developing non-addictive and anti-nociceptive drugs with less side-effects and KOR agonists have been reported to reduce addiction and affective disorders [32, 33]. Though KOR analgesics may lead to hallucinogenic side effects [34], plausible strategies have been utilized to reduce side effects including developing G-protein specificity agonists at KOR [35], or peripherally application KOR agonists, since most KOR-mediated side effects are resulted from systemic exposure [36].

Despite recent researches have confirmed a pivotal role of KOR in multiple pathologies including OA [37], the detailed molecular mechanism (s) are yet still to be elucidated. Our study demonstrated that activation of KOR by U50,488H local intra-articular delivery decreased pain perception and cartilage degeneration in DMM mice (Fig. 1), observations are consistent with previous study [12]. *Bileviciute-Ljungar et*. al reported a significant decrease of cartilage oligomeric matrix protein in animals treated with U50,488H, suggesting reduction of cartilage damage. The anti-inflammatory and chondroprotective effects of U50,488H were abolished by administration of the peripheral opioid receptor antagonist naloxone methiodide [12]. The in vitro results in our study showed that U50, 488H also reduced catabolic effects induced by TNF-α in chondrocytes (Figs. 2, 4). RT-qPCR results revealed that U50, 488H decelerated expression of *Il-6* and* Il-1β* (Fig. 4A), in line with other study [38]. However, inhibition of *ADAMTS* at mRNA level was modest, suggesting that U50,488H mainly regulated the expression level of MMPs (Fig. 2) to protect chondrocytes from inflammation in this OA model. The inhibition of collagen degradation in DMM model confirmed the effect of U50,488H on MMPs (Fig. 1). Dynorphin A, endogenous opioid peptides to KOR, was previously reported that inhibit *Mmp3, Mmp13* and *Adamts-4* and *Adamts-5* mRNA levels in cartilage explants treated by TNF-α [13]. And then, another study proved that attenuation of cartilage degeneration by the selective KOR agonist JT09 in a rat model of posttraumatic OA [14]. These results all proved that activation of KOR could attenuate cartilage degeneration in posttraumatic OA, suggesting that KOR may be a new disease-modifying target in OA treatment, in addition to pain management.

In order to explore the molecular mechanism of KOR signaling in OA and screen downstream molecular targets, RNA-seq analysis was performed in this study. Results showed the STAT3 signaling pathway was down-regulated by U50,488H administration in chondrocytes upon TNF-α stimulation and this result was validated by WB and IF outcomes (see Fig. 4). The phosphorylation level of STAT3 on TNF-α stimulation was significantly reduced (Fig. 4B). Besides, the inhibition of STAT3 signaling by static, a specifical inhibitor of STAT3 [29], led to a dose-dependently reduction in the expression of MMP3 and MMP13 (Fig. 4F) in TNF-α treated chondrocytes. But U50,488H could not reverse the effects of TNF-α on NF-κB, MAPK and AKT (Fig. 4B-E). Our observation was different from what *Shi *et al. recently reported [15], in which, the nucleus translocation of NF-κB was inhibited by KOR activation in macrophages in OA mice. This indicates that the impact of KOR signaling on down-stream reactions may vary, depending on cellular types, agonists’ characteristics and/or stage of the disease. As is known, OA pathology is largely modulated by immune response, and KOR may play a role in the interactions between immune cells and chondrocytes, suggesting further exploration in our future research.

To investigate the molecular interactions between KOR and STAT3, we constructed the plasmid of pLV-KOR-TurboID to find KOR-related interactors of proteins of interest screened by mass spectrometry. According to the results of mass spectrometry and WB/IF (see Figs. 4,5), activation of KOR could promote the binding of KOR and STAT3 and decrease the phosphorylation level of STAT3 induced by TNF-α. Thereby the nucleus translocation of STAT3 was reduced (Fig. 5). The transcriptional regulator STAT3 involves in multiple cellular functions with an important role in vertebrate development and regulation of inflammation and immunity [39]. STAT proteins contain an SH2 domain which recognizes the receptor phosphotyrosine [40]. Typically, when activated receptors undergo a conformational change, exposing the tyrosine terminal, the STAT proteins get recruited and phosphorylated through a receptor-connected JAK kinase or the directly by the receptor [41]. Then, p-STATs dissociate from the receptor, and translocate into nucleus as dimers to bind specific DNA sites and regulate corresponding cytokine-related gene transcription. According to previous researches, the negative regulation of the JAK-STAT signaling pathway mainly involve the cytokine signaling inhibitor (CIS/SOCS), protein inhibitor of activated STAT (PIAS), and protein tyrosine phosphatase (PTP) families, which block STAT signaling either by inhibiting JAK activity, or deconstructing STAT dimer, or dephosphorylating activated receptors [42, 43]. Inhibition of the STAT signaling pathway significantly decreased the expression of MMP13 and ADAMT-5 in chondrocytes [44], and it also modulated the IL-6-induced cartilage catabolism [19]. It has been demonstrated that U50,488H depressed inflammatory response and oxidative stress by inhibiting the expression levels of p-STAT3, instead of total STAT3 in rat hippocampus [45]. While the interaction location was not clear in that report [45]. In our study, this is the first time to prove that KOR could bind to STAT3 and sequester STAT3 in the plasma membrane (Fig. 5) of chondrocytes in OA, and this may explain why the expression of p-STAT3 was decreased. This outcome may elucidate a potentially novel molecular mechanism for the role of KOR in articular cartilage inflammation and disease. The schematic of possible interactions is presented in Fig. 6.

Our study has some limitations worth mentioning. Although we identified that activation of KOR decelerated post-traumatic osteoarthritis via binding to STAT3, it should be noticed that there might be additional pathways involved in the function of KOR in OA which await validation and the precise binding location site of KOR and STAT3 should be further clarified. Future work will focus on exploration on these problems.

In conclusion, our data showed that activation of KOR by U50,488H attenuated the progression of OA in mice and inhibited inflammation and the catabolic activities in cartilage, by down-regulating STAT3 pathway. Here, we proved that activation of KOR promoted the binding of STAT3 and KOR, leading to less nucleus translocation of STAT3, and decreased expression of inflammatory cytokines and MMPs. These results may reveal new therapeutic possibilities for KOR agonists and they bring new insights into OA therapeutic approaches.

### Supplementary Information

Supplementary Material 1.

## Data Availability

No datasets were generated or analysed during the current study.
