# Supplementary material for: Activation of kappa opioid receptor suppresses post-traumatic osteoarthritis via sequestering STAT3 on the plasma membrane
Source: Cell Commun Signal. 2024 Jun 18;22:335. doi: 10.1186/s12964-024-01709-4 (PMC11186255; doi:10.1186/s12964-024-01709-4)
Supplement: Supplementary file 1 — Supplementary Material 1. [file 12964_2024_1709_MOESM1_ESM.docx]

Below are the original images of all of the WB. The original images present protein makers and the size of the proteins.


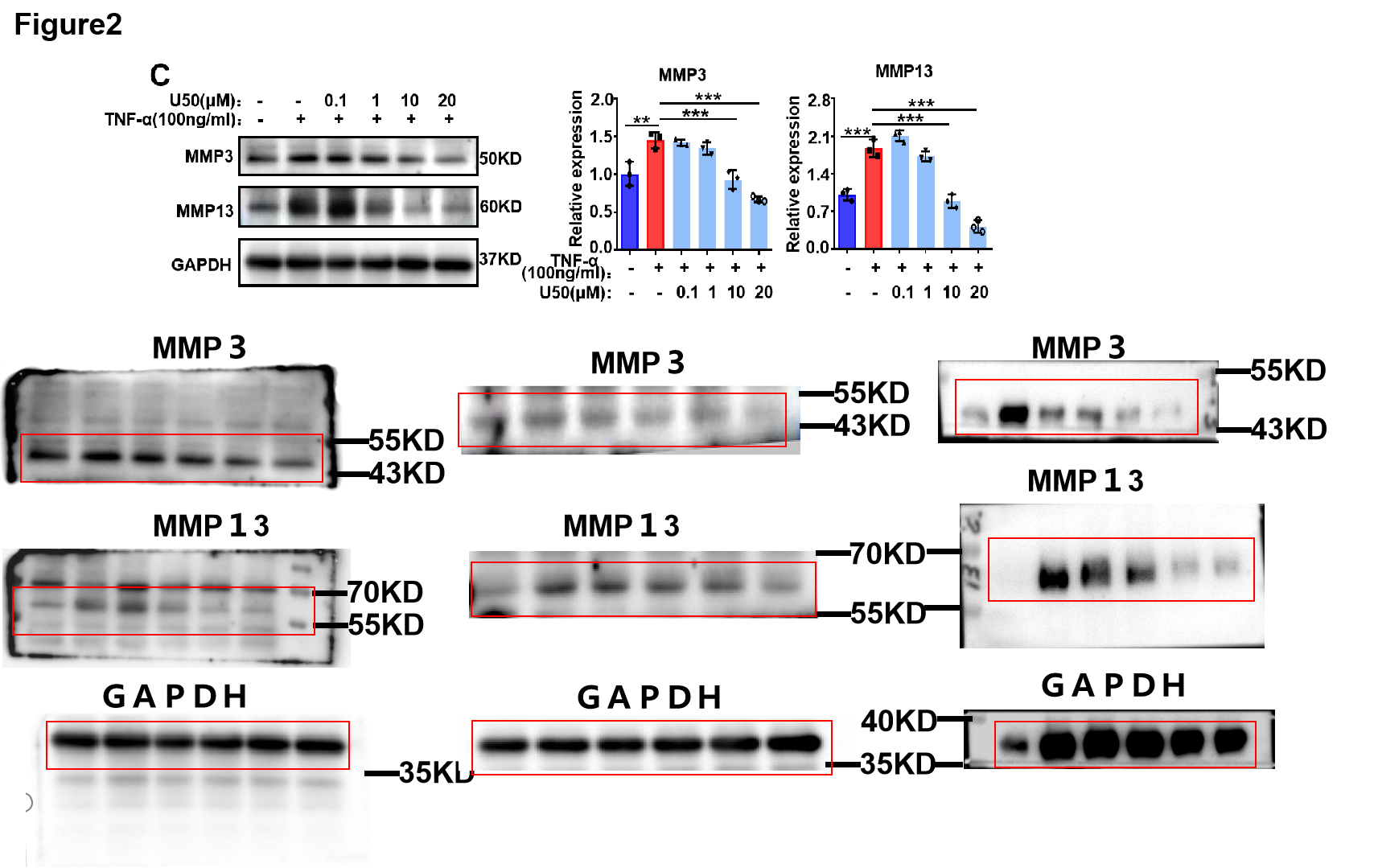


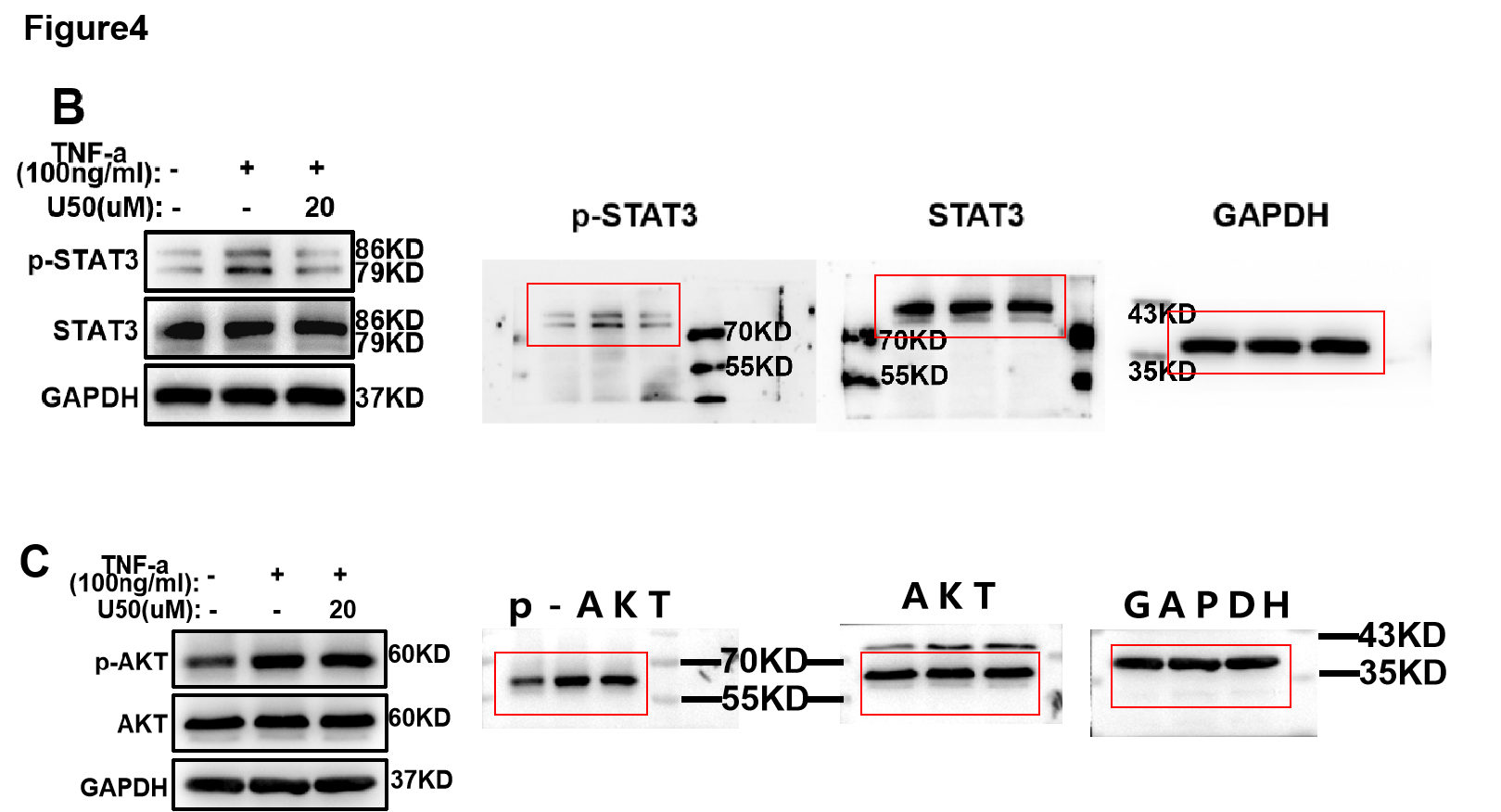

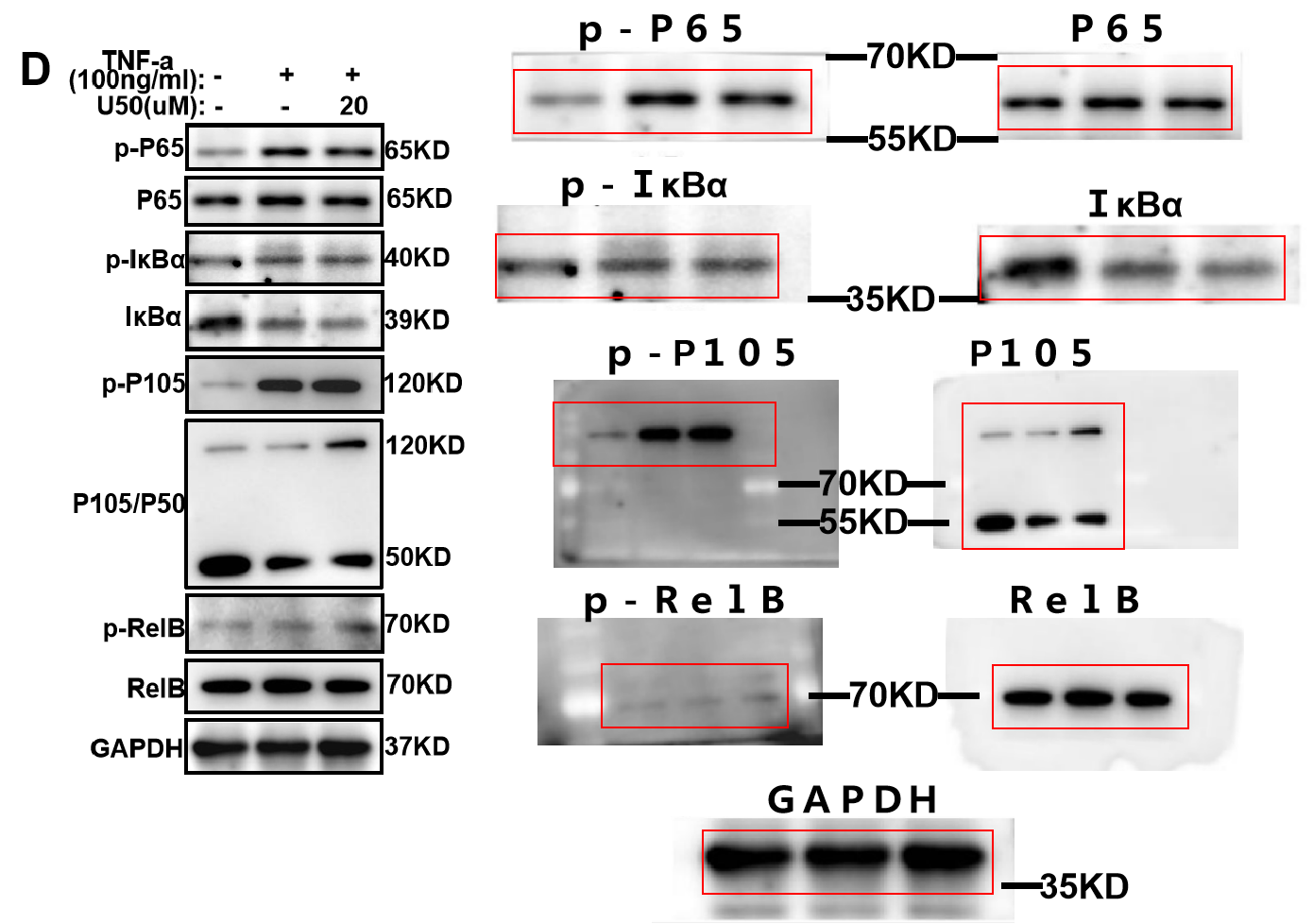


**130KD**


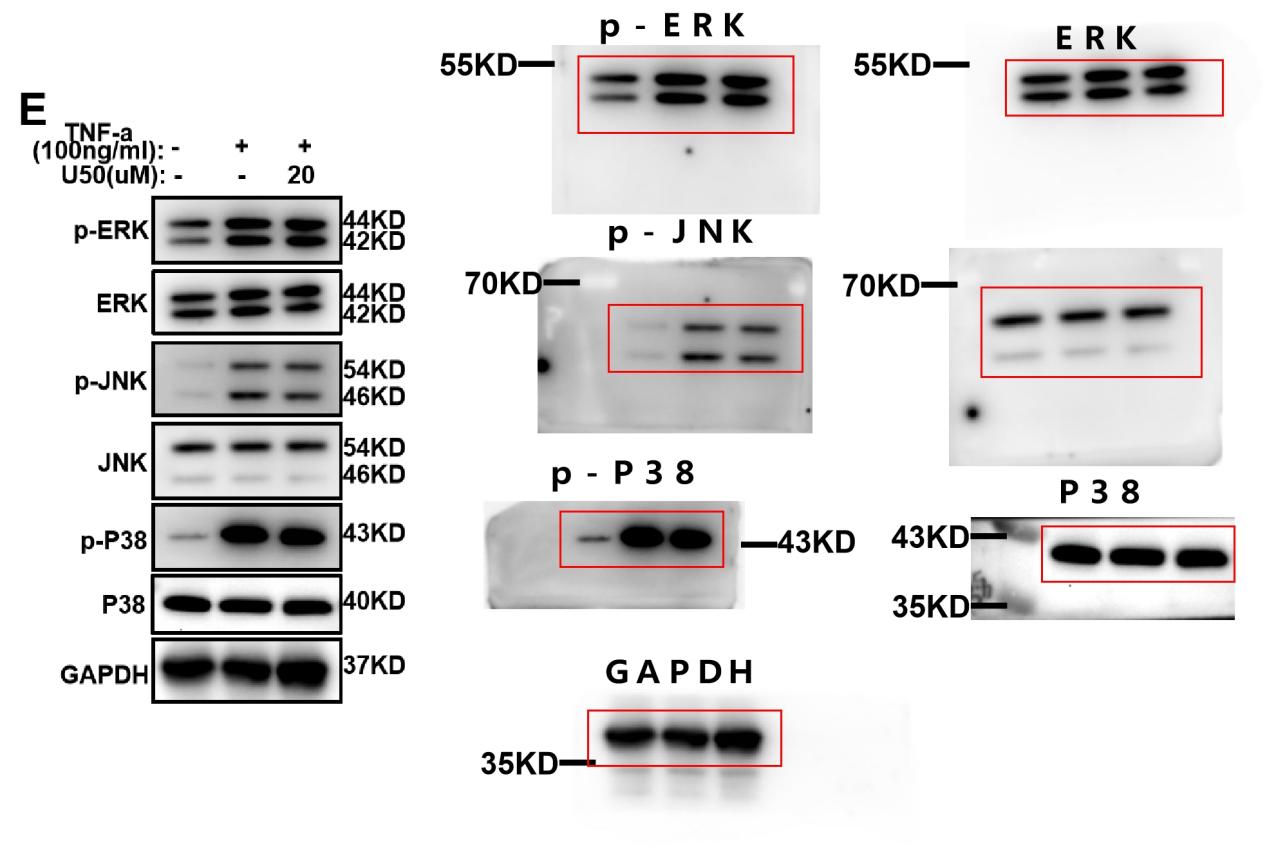


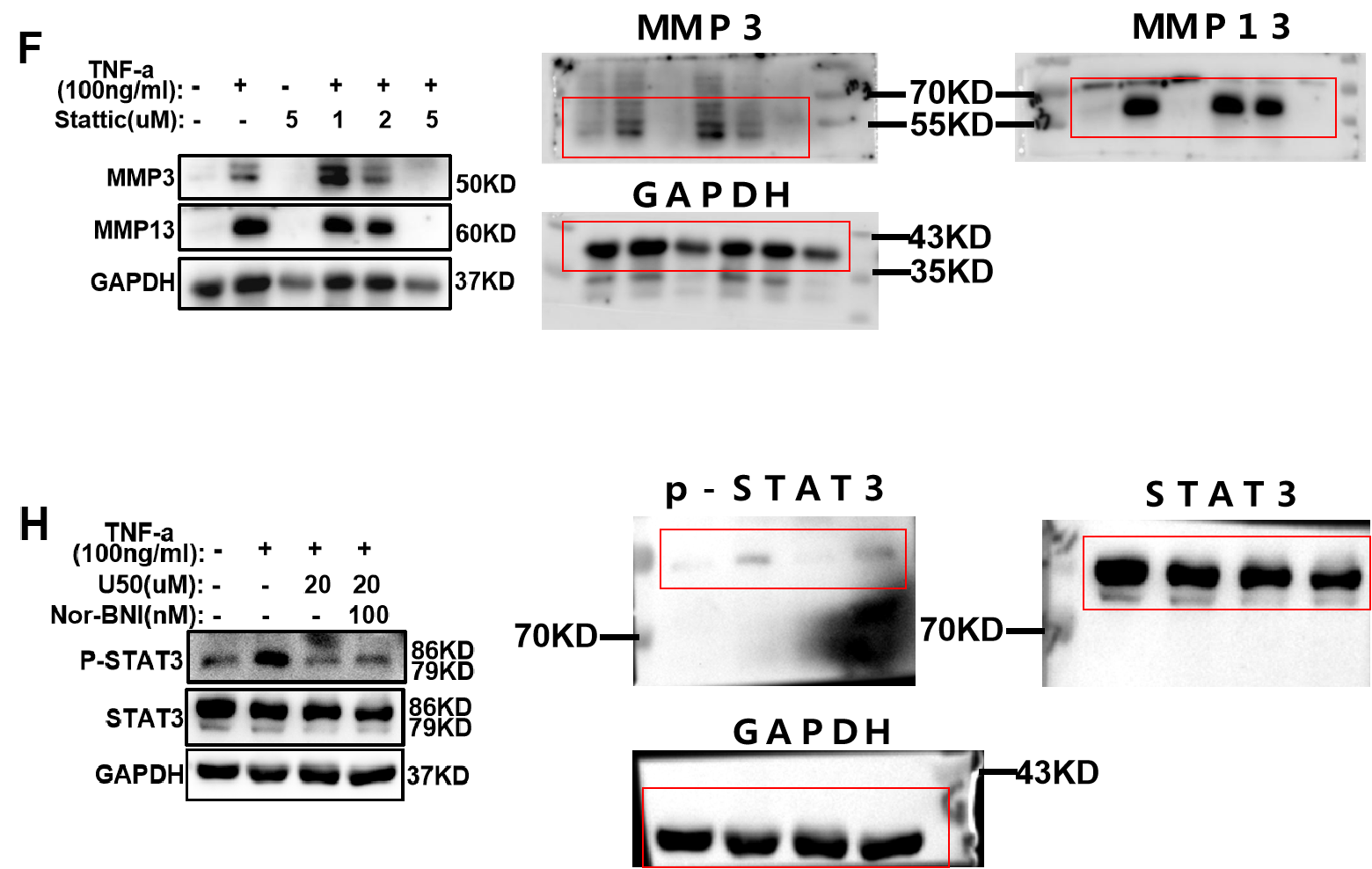


**Figure5**


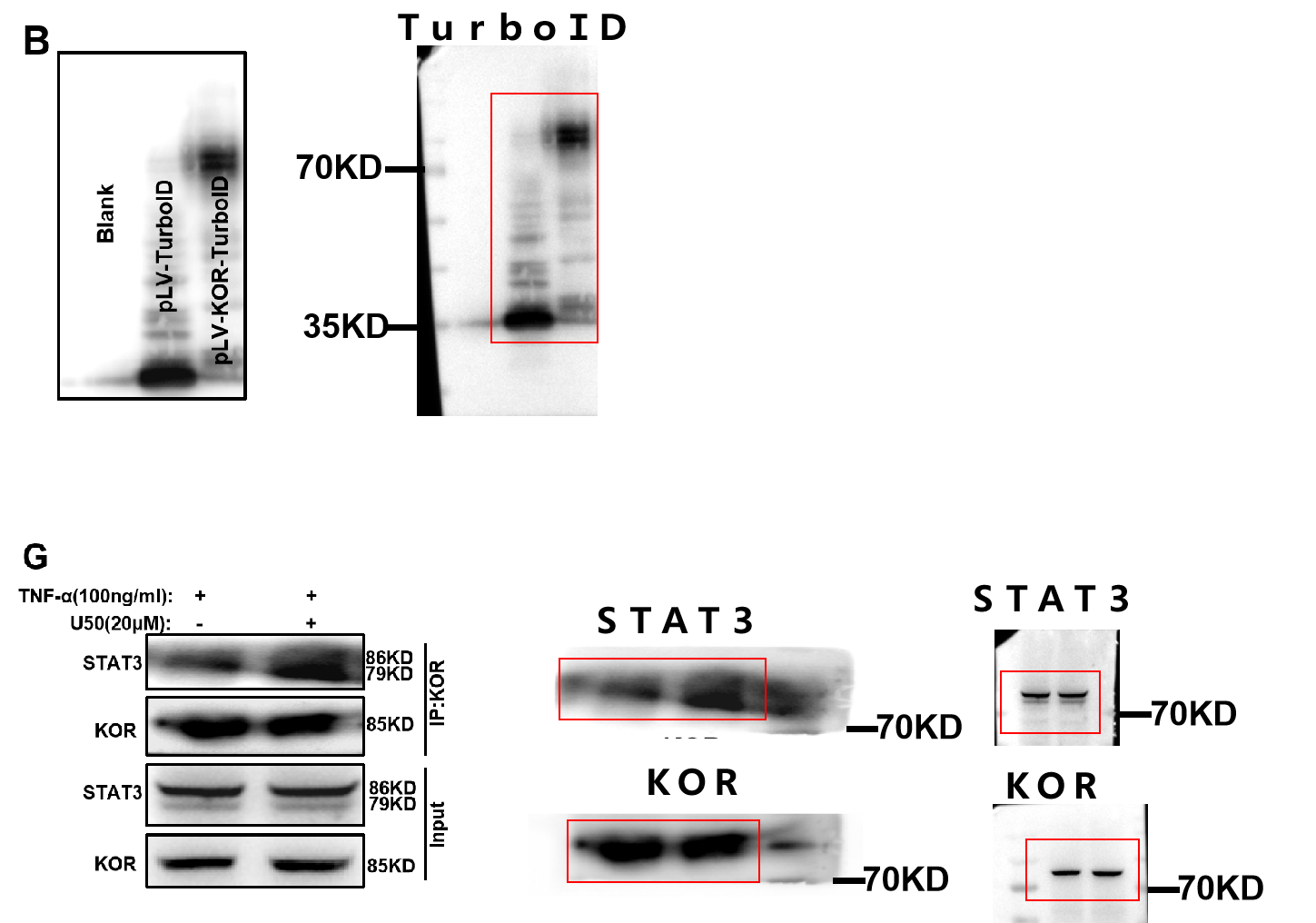

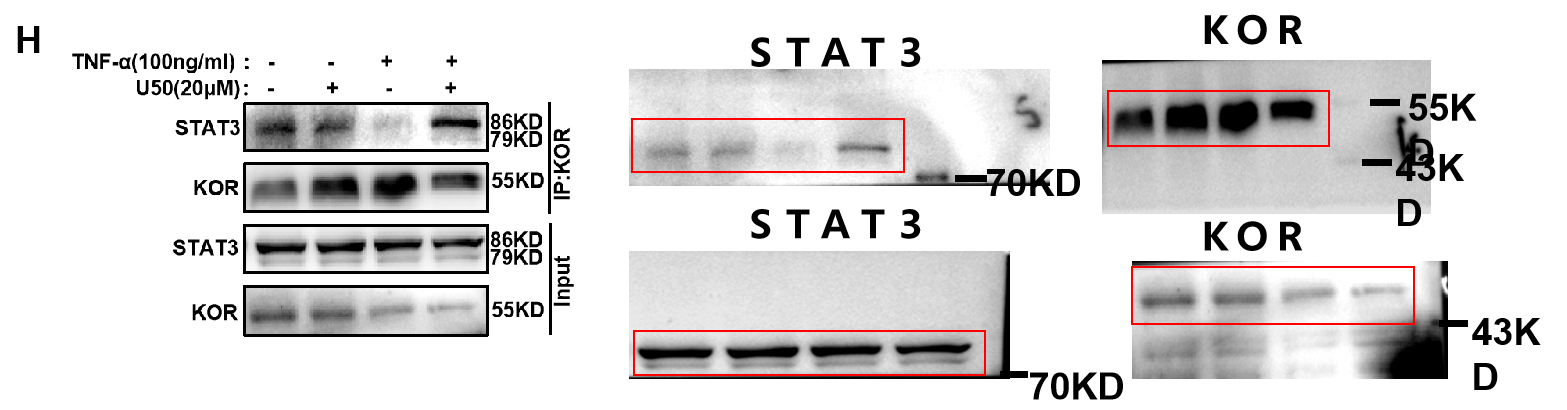


**95KD**

**95KD**

**130KD**

Figure 1. The original images presenting protein makers and the size of the proteins.

The below figure is immunofluorescence staining of STAT3 and KOR in tissues from joint. The results showed that STAT3 and KOR were mainly expressed in the layer of chondrocytes and relatively less in the subchondral layer of the joint.


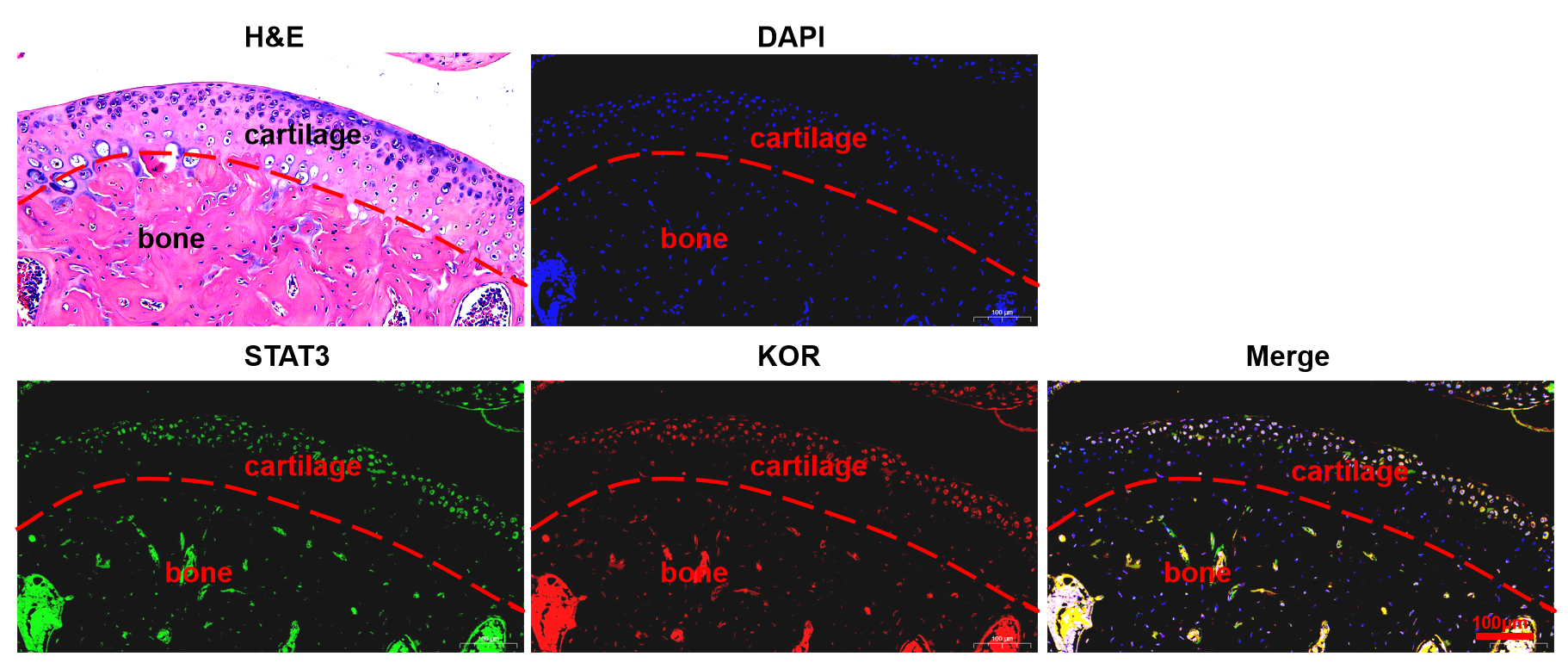


Figure 2. Immunofluorescence staining of STAT3 (ABclonal, China) and KOR in tissues from the mice model. Blue:DAPI, green: STAT3, red: KOR. Scale bar, 100 μm.
